# Exploring the use of accelerated intermittent theta burst stimulation combined with biofeedback based balance training in individuals with dementia and Alzheimer's disease

**DOI:** 10.1002/alz70858_102128

**Published:** 2025-12-25

**Authors:** Karishma R Ramdeo, Aimee J Nelson

**Affiliations:** ^1^ McMaster University, Hamilton, ON, Canada

## Abstract

**Background:**

Individuals with cognitive impairment are at an elevated risk of falls compared to those without. Impaired balance is a significant risk factor for falls, and emerging evidence suggests that balance control may serve as a marker of cognitive decline.

**Method:**

Repetitive transcranial magnetic stimulation (rTMS), a non‐invasive brain stimulation technique, has been shown to enhance synaptic plasticity, thereby improving motor and cognitive function. This study aimed to investigate whether an accelerated protocol of rTMS delivered over 14 days could improve cognition and balance in individuals with dementia and Alzheimer's disease. Participants were randomized into three groups: rTMS targeting the primary motor cortex (M1), the dorsolateral prefrontal cortex (DLPFC), or a placebo stimulation group. Accelerated intermittent theta burst stimulation (aiTBS) was applied daily, followed by 10 minutes of biofeedback‐based balance training. Balance training focused on improving center‐of‐pressure control, targeting left/right, front/back, and diagonal weight‐shifting abilities.

**Result:**

Preliminary results indicate that the M1‐targeted group showed significant improvements in balance, as measured by Limits of Stability and Balance and Fall Risk assessments and an improvement in cognition, as measured by the MoCA, compared to the DLPFC and placebo group.

**Conclusion:**

This study demonstrates the first application of aiTBS combined with balance training to enhance balance and cognition. These findings suggest the potential clinical utility of this combined approach for managing symptoms of dementia and Alzheimer's disease.

